# Identification of a novel B-cell epitope of the African swine fever virus p34 protein and development of an indirect ELISA for the detection of serum antibodies

**DOI:** 10.3389/fmicb.2023.1308753

**Published:** 2024-01-12

**Authors:** Yuanyuan Tian, Chao Liang, Jingming Zhou, Fanglin Sun, Yankai Liu, Yumei Chen, Xifang Zhu, Hongliang Liu, Peiyang Ding, Enping Liu, Ying Zhang, Sixuan Wu, Aiping Wang

**Affiliations:** ^1^Longhu Laboratory, Zhengzhou, China; ^2^School of Life Sciences, Zhengzhou University, Zhengzhou, China; ^3^College of Veterinary Medicine, Henan Agricultural University, Zhengzhou, China

**Keywords:** African swine fever virus, p34 protein, monoclonal antibody, B-cell epitope, antibody detection, ELISA

## Abstract

African swine fever (ASF) is a viral disease caused by the African swine fever virus that can be highly transmitted and lethal in domestic pigs. In the absence of a vaccine, effective diagnosis is critical for minimizing the virus’s spread. In recent years, with the decline of African swine fever virus (ASFV) virulence, antibody detection has become an important means of detection. ASFV nucleocapsid protein p34 is a mature hydrolytic product of pp220, which is highly conserved and has a high content in the structural protein of the virus. Prokaryotic cells were chosen to generate highly active and high-yield p34 protein, which was then used as an antigen for producing mouse monoclonal antibodies. The B-cell epitope ^202^QKELDKLQT^210^, which was highly conserved and found on the surface of the p34 protein, was first identified by an anti-p34 monoclonal antibody utilizing the peptide scanning technique and visualized in helix. This supported the viability of p34 protein detection even further. In addition, we established an indirect ELISA assay based on p34 to detect ASFV antibodies. The coincidence rate of this method with commercially available kits was shown to be 97.83%. Sensitivity analysis revealed that it could be detected in serum dilution as low as 1:6400, and there was no cross-reaction with other prevalent porcine epidemic diseases classical swine fever virus (CSFV), foot-and-mouth disease virus (FMDV), porcine reproductive and respiratory syndrome virus (PRRSV), and porcine circovirus 2 (PCV2). In summary, the established ELISA method and anti-P34 monoclonal antibody have demonstrated that the p34 protein has a promising application prospect for the detection of African swine fever antibodies.

## Introduction

African swine fever (ASF) is a highly contagious and fatal viral disease that may cause death in both domestic and wild pigs (Parker et al., 1969; Thomson et al., 1980; Anderson et al., 1998). ASF emerged in Kenya first (Montgomery et al., 1921) and remained endemic in Africa until it spread to Europe in the middle of the last century and later to South America and the Caribbean. In the 1990s and early 2000s, the epidemiology and distribution changed: African swine fever virus (ASFV) spread to other areas not normally affected by African swine fever, including Cote d ‘Ivoire (1996), Nigeria (1997), Togo (1997), Ghana (1999), Burkina Faso (2003), and recently, Chad (2010). ASFV has also spread to some islands such as Madagascar (1998) and Mauritius (2007). Vitally, the disease spread again from Africa to the Caucasus region of Georgia in 2007 and in 2014 to the eastern territories of the European Union. By 2018, it had spread to China, and rapidly occupied much of Southeast Asia and Oceania (Kolbasov et al., 2018). For the first time, many countries are experiencing non-plague outbreaks, including Hungary (2018), Bulgaria (2018), Slovakia (2019), Serbia (2020), Greece (2020), Mongolia (2019), Vietnamese (2019), Korea (2019), and so on. Due to globalization, the African swine fever virus has become highly resistant to the environment and meat, and there is a lack of effective vaccine control. As a result, the incidence of African swine fever has increased in endemic countries and has even spread to previously disease-free territories over the past 15 years.

Responsible for massive losses in pig populations and drastic economic consequences, African swine fever (ASF) has become a major crisis for the pork industry. Researchers have continuously conducted studies on African swine fever vaccines. Traditional vaccines have not provided complete protection, but various other types of vaccines have been explored, such as inactivated vaccines, live attenuated vaccines, subunit vaccines, DNA vaccines, and virus-vectored vaccines (Lim et al., 2023). The latest research findings on the recombinant live attenuated vaccine ASFV-G-ΔI177L/ΔLVR offer promising prospects for the development of a safe and effective vaccine (Urbano and Ferreira, 2022). At present, due to the lack of a protective vaccine and effective treatment, the most effective way to prevent and control ASF is currently to trap and kill infected pigs.

Early diagnosis of infected pigs is the most effective way to control the spread of the virus. A positive serological antibody test for ASFV confirms ongoing or past infection. Typically, antibodies in surviving pigs persist for months or years after acute or subacute infection. Thus, antibody detection can be used for large-scale screening of non-infected or chronic animals. The ELISA method is effective in detecting specific antibodies in pigs after infection with ASFV (Kolbasov et al., 2018) and the most commonly used antigen targets for clinical detection are p30, p54, and p72 (Cao et al., 2021; Yu et al., 2021; Geng et al., 2022). However, other antigenic targets still need to be developed and explored.

ASF is caused by ASFV, a large double-stranded nucleocytoplasmic DNA arbovirus, the only member of the Asfarviridae family (Dixon et al., 2013), with an icosahedral morphology and an average diameter of 250 nm. The viral genome consists of a single molecule of linear, covalently closed, double-stranded DNA. The genome length of different strains ranges from 170 ~ 190 kbp (Dixon et al., 2013) and encodes 151–167 open reading frames (ORFs) that can encode more than 150 polypeptides (Carrascosa et al., 1985; Esteves et al., 1986; Yáñez et al., 1995), encoding 54 structural proteins and about 100 polypeptides (Malmquist and Hay, 1960; Salas and Andrés, 2013). The main component of the viral capsid, protein p72, the two structural proteins p30 (p32) and p54, and the multiprotein pp62, have been identified as the most antigenic of the proteins responsible for the induction of antibodies after natural infection (Pastor et al., 1989; Gallardo et al., 2006) and are important as serodiagnostic targets. A distinctive feature of ASFV gene expression is the synthesis of two polyprotein precursors, called pp220 and pp62, to produce several major components of the viral particle. The polyprotein pp220, encoded by the CP2475L gene, is an N-carnosylated polypeptide that is processed by protein hydrolysis to produce the proteins p150, p37, p34 and p14 (Simón-Mateo et al., 1993). These proteins are present in equal molecular quantities in mature viral particles and account for about a quarter of their total protein mass (Andrés et al., 1997). Among them, the p34 protein is abundant and highly conserved in all virulent strains and is a promising antigenic target as a test.

The p30, p54, and p72 proteins usually need to be expressed in the eukaryotic system to obtain post-translational modifications, and the expression amount is not very ideal, which increases the difficulty of antigen acquisition to a certain extent. The p34 protein has been proven to have a good reaction effect with positive serum and is a highly conserved nucleocapsid protein, which is easy to obtain *in vitro*, that is, the prokaryotic system can obtain a large number of proteins with good activity, saving costs to a certain extent. Herein, we used the prokaryotic system to prepare a large number of highly active p34 recombinant proteins and monoclonal antibodies against p34. Based on this, we established an indirect ELISA method, which provides a new ideal antigen target and application prospect for ASFV antibody detection.

## Materials and methods

### Cells and serum samples

The *Escherichia coli Trans* 5α and *Trans* BL21(DE3) competent cells were purchased from TransGen Biotech (Transgen, Cat. No. CD201-01, CD601-02, China). Hybridoma cells SP2/0 were purchased from ATCC (Manassas, Cat. No. BFN60806599, United States). African swine fever virus (ASFV) standard-positive serum, ASFV-negative serum, and other porcine disease-positive sera were purchased from the China Veterinary Culture Collection Center. Positive samples, which were verified by qRT-PCR, were preserved in our laboratory.

### Expression and purification of p34 protein

The gene encoding ASFV p34 protein (Pig/HLJ/2018, Genbank Access Number: MK333180.1) was codon optimized, inserted into the pCAGS3 vector, and synthesized by Sangon Biotech (Shanghai) Co., Ltd. A pair of primers (Table 1) was designed to amplify the p34 protein by adding the *BamHI/XhoI* enzyme cutting sites and cloned to the pET-32a vector for expression in *Escherichia coli*. BL21(DE3) cells. Specifically, recombinant expression bacteria pET-32a-34-BL21(DE3) were induced at 0.5 mM isopropyl-β-D-thiogalactopyranoside (IPTG) at 16°C for 10 h. Proteins were collected by centrifugation at 12000 rpm for 5 min, sonicated total for 15 min (ultrasound 3 s and stop 5 s), supernatant was collected by centrifugation at 12000 rpm for 20 min, and the supernatant was collected using HisTrap™ excel column (GE, Cat No. 10291104, Sweden) at 20–200 mM imidazole (20 mM Tris, 150 mM NaCl). The expression of the p34 protein was verified via Western blot by His-Tag and ASFV standard positive sera, and purification was determined by SDS-PAGE.

**Table 1:** Primers used in this study.

Name	Sequence (5′-3′)	Position (aa)
p34-F	CGC *GGATCC* ATGGATAAGAACCCTGTGCAG	1–324
p34-R	CCG *CTCGAG* TTAATCGCCGCCCTTCTTAGC	
p34-1F	CGC *GGATCC* ATGGATAAGAACCCTGTGCAG	1–60
p34-1R	CCC *AAGCTT* TTATCTAGGATCAGGCAGCTG	
p34-2F	CGC *GGATCC* ATGGATATCAAGAAGCAGCTG	51–110
p34-2R	CCC *AAGCTT* TTACACGATCTGTCTACAGAT	
p34-3F	CGC *GGATCC* ATGGGCGCCGCCTCTATCTGT	101–160
p34-3R	CCC *AAGCTT* TTAGGCGGCCTTTGTCACCTC	
p34-4F	CGC *GGATCC* ATGAGAATGGTGACCGAGGTG	151–210
p34-4R	CCC *AAGCTT* TTATGTCTGCAGCTTATCCAG	
p34-5F	CGC *GGATCC* ATGACACAGAAGGAGCTGGAT	201–260
p34-5R	CCC *AAGCTT* TTAGCCCACCTTCTGCAGAGC	
p34-6F	CGC *GGATCC* ATGAAGATCAACAAGGCTCTG	251–324
p34-6R	CCC *AAGCTT* TTAATCGCCGCCCTTCTTAGC	
p34-4-1F	CGCGGATCCATGAGAATGGTGACCGAGGTG	151–180
p34-4-1R	CCCAAGCTTTTACAGCAGCCTTCTGTACAC	
p34-4-2F	CGCGGATCCATGAACGAGCAGAACCTGCAG	181–210
p34-4-2R	CCCAAGCTTTTATGTCTGCAGCTTATCCAG	

### Generation of monoclonal antibodies against p34 protein

The generation procedures of monoclonal antibodies were according to the previous study (Tian et al., 2022). Briefly, 6–8 weeks old BALB/C mice were immunized with 20 μg of purified p34 protein in Freund’s complete adjuvant (Sigma-Aldrich, Cat No. F5881, USA) per mouse injection on the back. After a 3-week interval, booster immunization was performed with 20 μg of purified p34 protein in Freund’s incomplete adjuvant (Sigma-Aldrich, Cat No. F5506, USA). Antibody titers of anti-p34 protein in mouse serum were measured after 2 times booster immunization, and the highest titers of the mouse were donated spleen for cell fusion. The mouse myeloma cells SP2/0 and the spleen cell suspension were fusion by Polyethylene Glycol 1,500 (Sigma-Aldrich, Cat No. 10783641001, USA) in the RPMI 1640 (Solarbio, Cat No. 31800, China) with hypoxanthine–aminopterin–thymidine supplement (HAT) (Sigma-Aldrich, Cat No. H0262, USA) for screening hybridoma cells. Monoclonal cells were obtained by ELISA screening and dilution method (Tian et al., 2022).

### Characterization of monoclonal antibody against p34

A large amount of monoclonal antibody 1E6 was obtained by inducing ascites *in vivo* and purified by Protein A column according to the manufacturer’s instructions (GenScript, Cat No. L00210, China). The ELISA was used to analyze the affinity and titers of mAb 1E6 using p34 protein as the coating antigen. The affinity parameter “Ka” was calculated according to the previous research (Tian et al., 2022). The subtypes of mAb 1E6 were determined by the Mouse Monoclonal antibody Subtype Identification kit (Proteintech, Cat No. PK20002, USA).

### Mapping the B-cell epitope recognized by the mAb against p34

The B-cell epitope recognized by the mAb 1E6 was determined by the ELISA and dot-blot assay. The whole length of the p34 protein was truncated into six fragments and 60 amino acid (aa) residues per fragment with 10 aa overlapped between adjacent fragments. The truncated fragments were designed to be inserted into the prokaryotic expression vector pET-32a and expressed in the *E. coli* BL21(DE3) cells induced by 0.5 mM IPTG. Based on the results of the recognition of truncated fragments by mAb 1E6, primers were continued to be designed for more accurate truncation. Primers used in truncated segments design are shown in Table 1. Finally, peptide sequences of 12 amino acids in length were synthesized for B-cell identification.

### Establishment and optimization of p34 ELISA

The established ELISA method was optimized in the following aspects. The concentration of coating antigen p34 and the dilution of serum (primary antibody) were determined by checkerboard titration. Among them, concentrations of p34 protein included 4 μg/mL, 2 μg/mL, 1 μg/mL, 0.5 μg/mL, 0.25 μg/mL, and 0.125 μg/mL and serum samples diluted with PBS at 1:50, 1:100, 1:200, and 1:400. The coating condition of p34 protein (37°C for 1 h, 37°C for 2 h or 4°C overnight) used carbonate buffer solution (0.05 M CBS, pH 9.6), sealing condition (5% skim milk in PBST (PBS containing 0.05% Tween-20) or 5% BSA), sealing time (37°C for 2 h, 4°C overnight) were optimized. The concentration of secondary antibody (HRP-conjugated Goat Anti-Swine IgG(H + L), Proteintech, Cat No. SA00001-5, USA) was then optimized (1:5000, 1:10000, 1:15000, 1:20000, 1:30000, and 1:40000). In addition, the incubation time of primary and secondary antibodies (20 min, 30 min, 45 min, and 60 min) and color development time (5 min, 10 min, 15 min, and 20 min at RT or 37°C) by TMB were also optimized. According to the ratio of OD450 value between positive and negative serum (P/N), the maximum ratio of P/N was regarded as the optimal reaction condition and chosen for subsequent ELISA assays.

### Application of the ELISA for detection of ASFV p34

Swine serum samples (*N* = 78) were used to analyze the conformance between the established ELISA assay and the commercially available commercial kit (Shanghai Jianglai Biological Co., LTD, Cat No. JL47607). The sensitivity of the ELISA method established was analyzed by ASFV standard positive and negative sera (diluted at 1:100, 1:200, 1:400, 1:800, 1:1600, 1:3200, 1:6400, 1:12800, 1:25600, and 1:51200). The specificity of the ELISA assay established in this study was tested using the standard positive serum of ASFV, classical swine fever virus (CSFV), foot-and-mouth disease virus (FMDV), porcine circovirus 2 (PCV2), and porcine reproductive and respiratory syndrome virus (PRRSV). The intra-assay repeatability was verified by repeating the experiment three times at the same time, and inter-assay repeatability was determined by three independent coated ELISA plates for the same samples.

### Statistical analysis

Each experiment was independently repeated 3 times. Statistical significance was considered at a value of *p* of <0.05. All data analyses used the software GraphPad Prism (Version 9.5.1) (GraphPad Software, San Diego, California, USA).

## Results

### Preparation of recombinant protein p34

**Figure 1:**
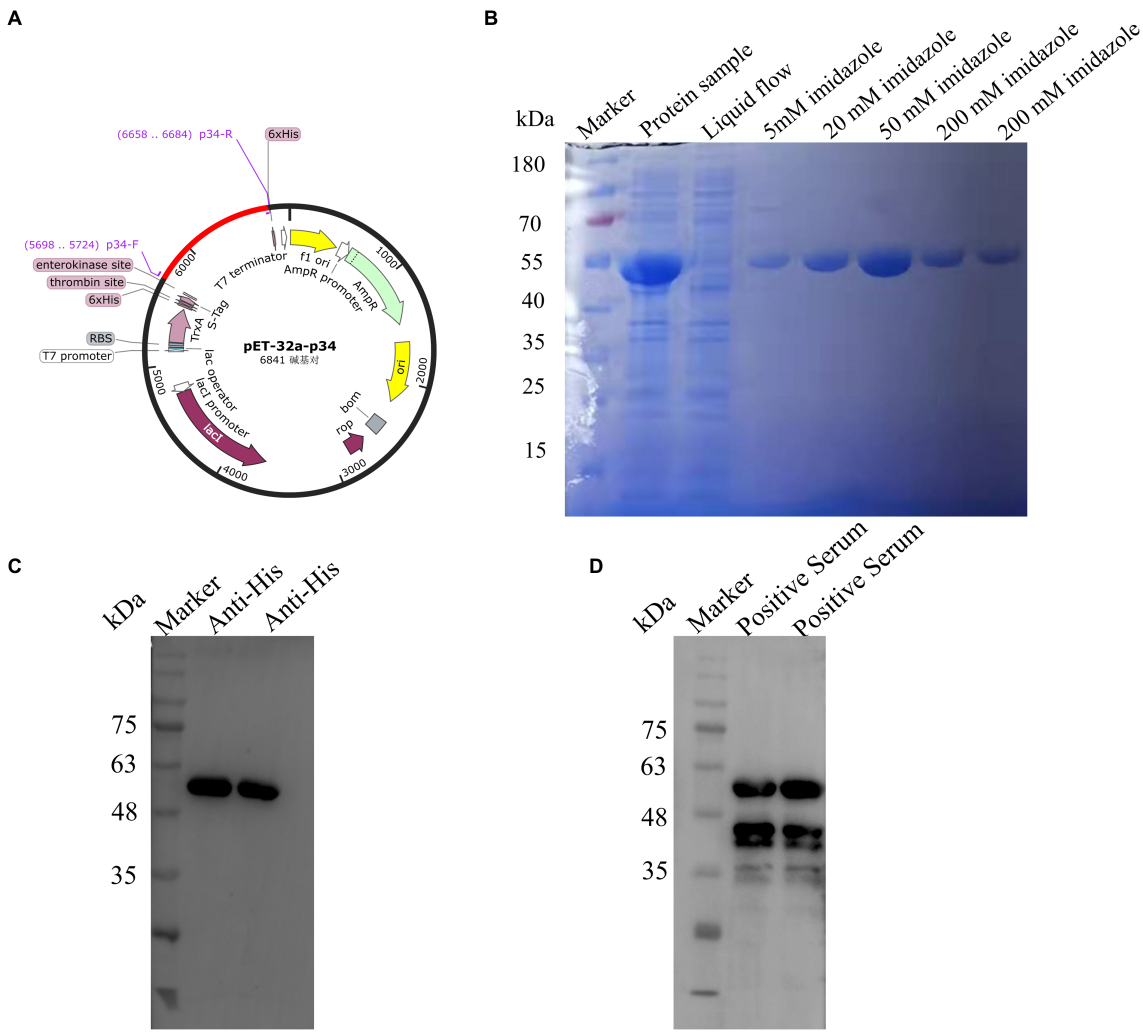
Construction and expression of African swine fever p34 protein. **(A)** The p34 antigen protein was designed according to the molecular biological guidelines. **(B)** SDS-PAGE was used to verify the purification results of the pET-32a-34 recombinant protein. **(C)** Western blot assay was used to verify that the pET-32a-34 protein could react with His mAb, indicating the successful expression of the p34 recombinant protein. **(D)** Western blot assay was used to verify the reaction of pET-32a-34 protein with ASFV-positive serum, confirming the antigenic activity and correct expression of the p34 recombinant protein.

The full length of encoding p34 protein gene was amplified and constructed on pET-32 vector for expression according to standard molecular construction principles (Figure 1A). The p34 protein was expressed in soluble form in *E. coli* BL21(DE3) cells, and obtained with a purity of approximately 95% by Ni affinity chromatography (Figure 1B). The purified p34 protein could bind to (6×) His mAb (Figure 1C) and the ASFV standard positive serum, which also verified the correct expression and protein activity of p34 protein (Figure 1D), the size of which was in accordance with the theoretical value.

### Preparation of monoclonal antibodies against p34 protein

**Figure 2:**
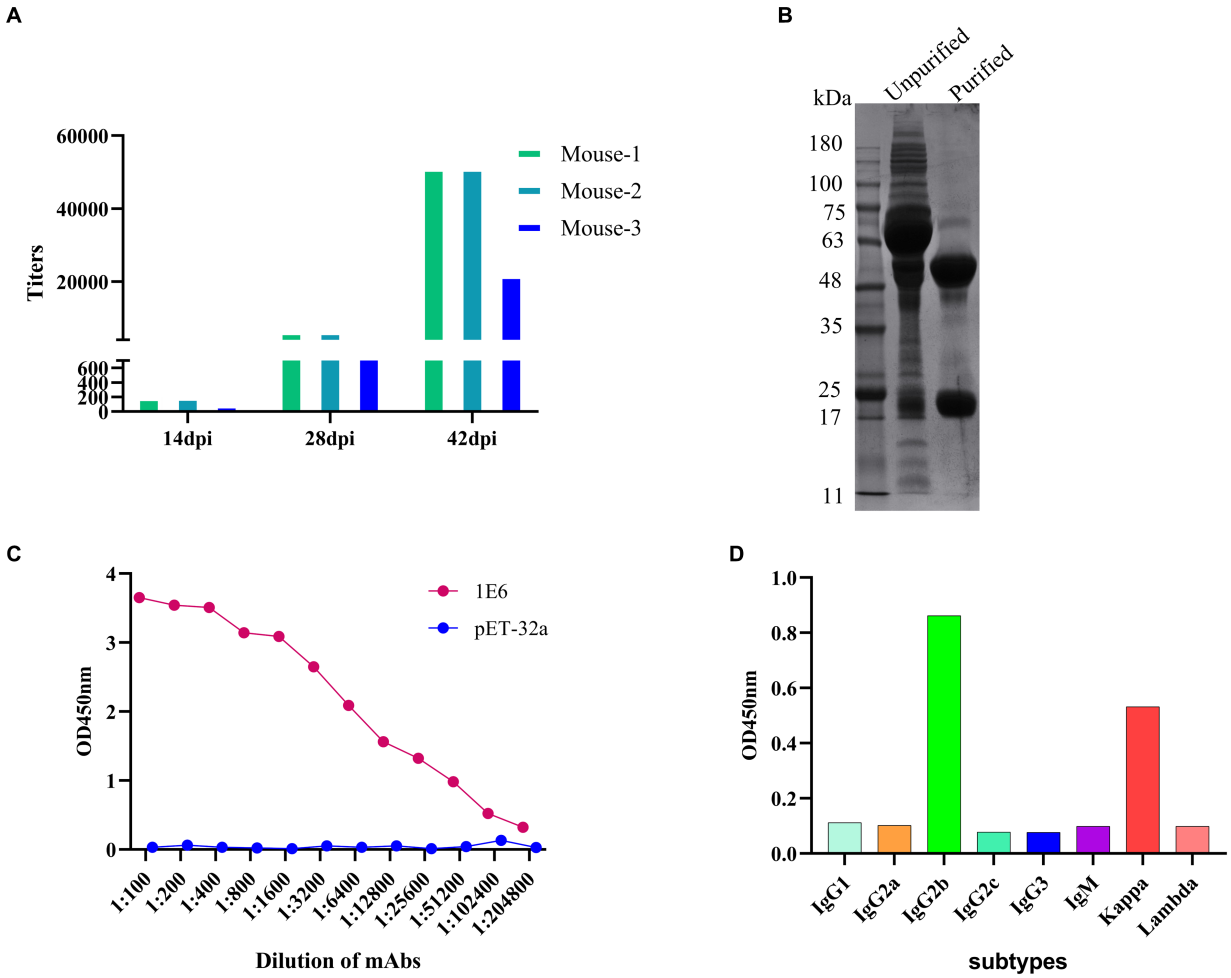
Preparation and characterization of anti-P34 monoclonal antibody. **(A)** Determination of anti-p34 serum titer in immunized BALB/c mice. **(B)** The SDS-PAGE showed the result that the mAb 1E6 was purified via Protein A affinity chromatography. **(C)** The titer of purified mAb 1E6 was determined by I-ELISA (p34 as the coating antigen, pET-32a as the negative control coating antigen). **(D)** Subtype determination of monoclonal antibody 1E6.

BALB/C mice were immunized according to standard immunization procedures. After booster immunization, the antibody titers of anti-p34 protein in mice could reach 1:51200 (Figure 2A), and the Mouse-1 with the highest titer was selected for cell fusion, and the 1E6 monoclonal cell line was obtained via ELISA screening and subcloning. Monoclonal antibody 1E6 was prepared using the *in vivo* mouse-induced ascites method and purified by a Protein A column (Figure 2B). The titer of mAb 1E6 was as high as 1:204800, as measured by the i-ELISA (Figure 2C). The monoclonal antibody 1E6 has a high affinity, with an affinity constant at 4.3 × 10^8^ L/mol. The light chain of mAb 1E6 was kappa and the heavy chain was IgG2b, as determined by the subtype kit (Figure 2D).

### Mapping the B-cell epitope recognized by the mAb against p34

**Figure 3:**
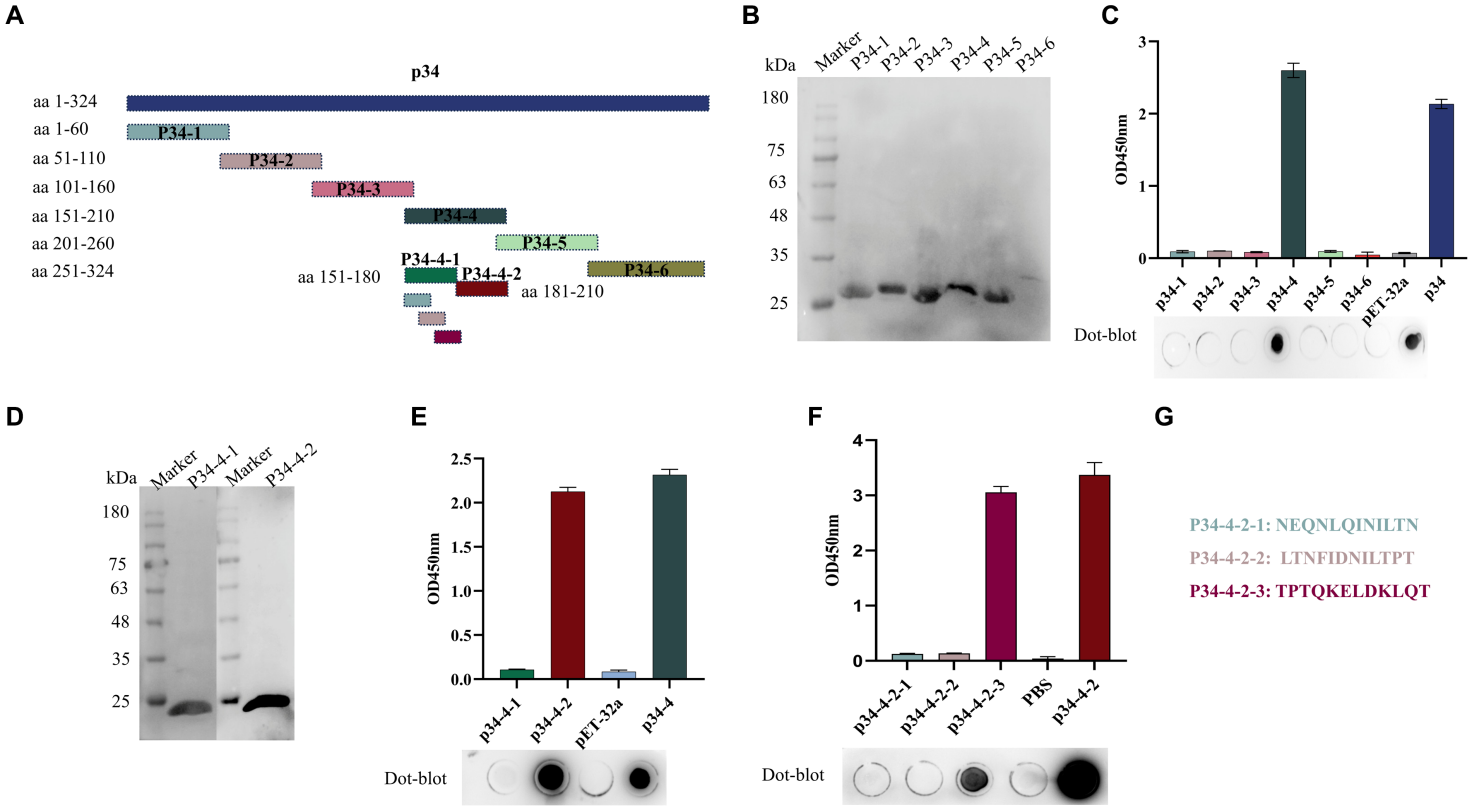
Identification and mapping of epitope regions by mAb 1E6. **(A)** Truncation strategy for mapping the epitope. **(B)** Western blot was used to confirm that the truncated expression fragments of p34 (p34-1, p34-2, p34-3, p34-4, p34-5, and p34-6) could be detected by His mAb tag. **(C)** I-ELISA and dot-blot assay were used to determine the region recognized by the mAb 1E6. **(D)** Western blot was used to confirm that the precise truncated expression fragments of p34-4 (p34-4-1 and p34-4-2) could be detected by His mAb tag. **(E)** I-ELISA and dot-blot assay were used to determine the precise region recognized by the mAb 1E6. **(F)** Map the B-cell epitope of the p34 protein using the mAb 1E6. **(G)** The sequence of synthetic peptides used in this research.

We identified and analyzed the B-cell epitope recognized by the mAb against p34 in order to better validate the crucial region where mAb 1E6 operates. We designed primers to truncate the p34 protein into short fragments (Figure 3A), and then react with mAb 1E6 to obtain key action regions. First, six truncated fragments could react with His mAb by Western blot for the verification of the successful expression (Figure 3B). The results of dot-blot and ELISA showed that the 1E6 can react with the p34-4 (aa 151–210) (Figure 3C). Further to truncate the p34-4 into p34-4-1 and p34-4-2 (Figure 3D), 1E6 could bind with the p34-4-2(aa 181–210) (Figure 3E). In order to more precisely analyze the sequence of epitopes specifically bound by mAb 1E6, three peptides were synthesized (Figure 3G). Results of dot-blot assay and ELISA verified that aa ^202^QKELDKLQT^210^ was a B-cell epitope specifically recognized by the mAb 1E6 (Figure 3F).

### Bioinformatic analysis of linear B-cell epitope revealed the antigenicity and conservatism of epitopes

**Figure 4:**
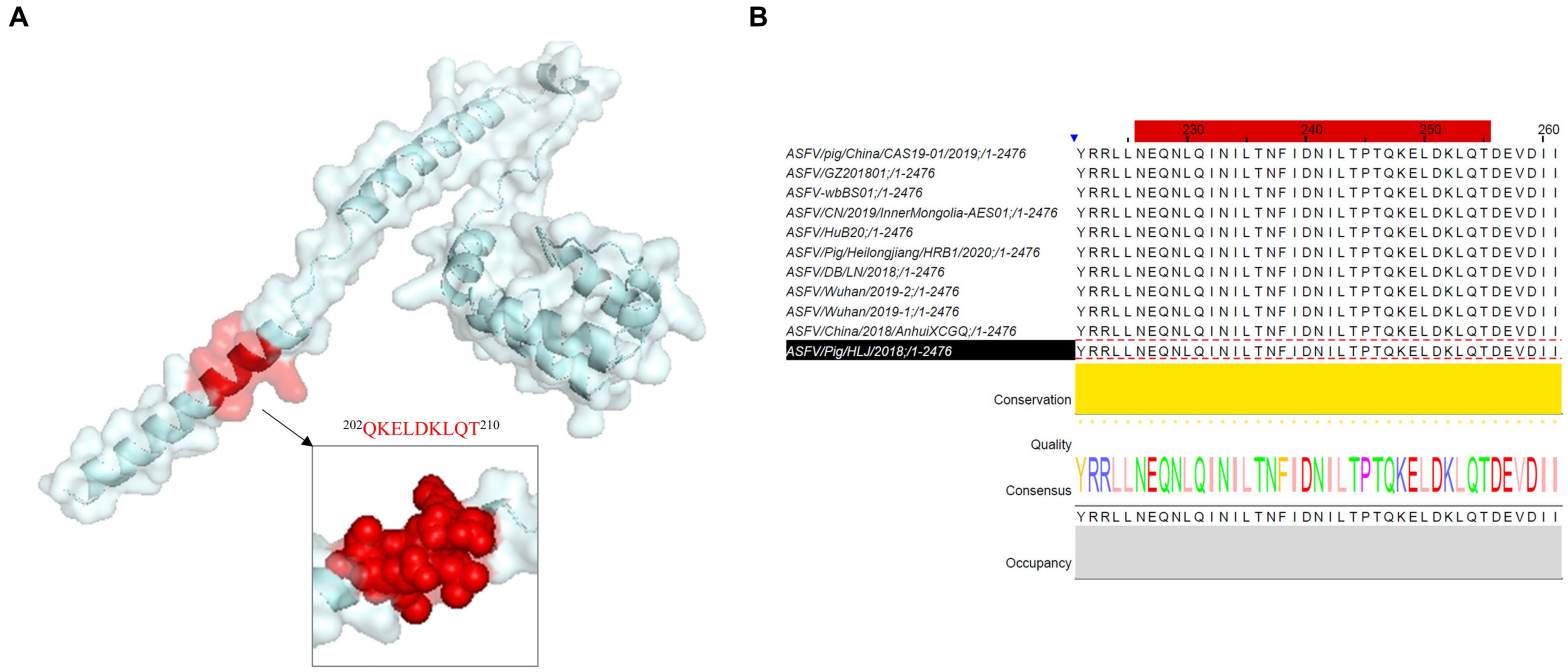
Spatial structure and conservation analysis of novel B-cell epitope identified in this research. **(A)** PyMOL software was used to visualize the position and form of the epitope region recognized by mAb 1E6 in the spatial structure. **(B)** Conservatism analysis of epitope identified in this study.

The B-cell epitope of p34 recognized by mAb 1E6 was analyzed by the software PyMOL and Jalview. The PyMOL software can be used to visualize the spatial position of epitopes, as shown in Figure 4A. The epitope was located on the spatial surface and visualized in helix form, which can further explain its strong antigenicity. As shown in Figure 4B, we analyzed the conservatism of epitopes by Jalview; the epitope “^202^QKELDKLQT^210^” was at a 100% conservative rate (Table 2), which further verified the feasibility and advantages of p34 protein and monoclonal antibody for detection.

**Table 2:** Optimal dilutions of antigen coating and serum samples for ELISA developed in this study.

Serum dilution	Concentration of antigen coating (μg/ml)					
(×)	0.125	0.25	0.5	1	2	4
50(+)	0.855 ± 0.041	1.060 ± 0.038	1.169 ± 0.004	1.198 ± 0.046	1.194 ± 0.036	1.210 ± 0.013
50(−)	0.062 ± 0.033	0.067 ± 0.021	0.085 ± 0.012	0.079 ± 0.033	0.080 ± 0.027	0.078 ± 0.022
P/N	14.274	15.820	13.752	15.164	14.925	15.512
100(+)	0.778 ± 0.013	0.893 ± 0.027	1.010 ± 0.022	1.001 ± 0.031	1.005 ± 0.021	1.021 ± 0.043
100(−)	0.055 ± 0.027	0.062 ± 0.042	0.080 ± 0.016	0.072 ± 0.027	0.062 ± 0.038	0.067 ± 0.026
P/N	14.145	14.403	12.625	13.902	16.209	15.238
200(+)	0.659 ± 0.036	0.839 ± 0.045	0.870 ± 0.03	0.876 ± 0.052	0.865 ± 0.024	0.882 ± 0.025
200(−)	0.055 ± 0.004	0.056 ± 0.031	0.068 ± 0.011	0.062 ± 0.005	0.061 ± 0.015	0.063 ± 0.036
P/N	11.981	14.982	12.794	14.129	14.180	14
400(+)	0.494 ± 0.021	0.577 ± 0.024	0.689 ± 0.014	0.710 ± 0.045	0.708 ± 0.043	0.724 ± 0.021
400(−)	0.058 ± 0.012	0.058 ± 0.011	0.075 ± 0.003	0.062 ± 0.023	0.061 ± 0.028	0.062 ± 0.042
P/N	8.517	9.948	9.18	11.451	11.60	11.677

### Establishment and optimization of ELISA method for antibody detection of ASFV based on p34

**Figure 5:**
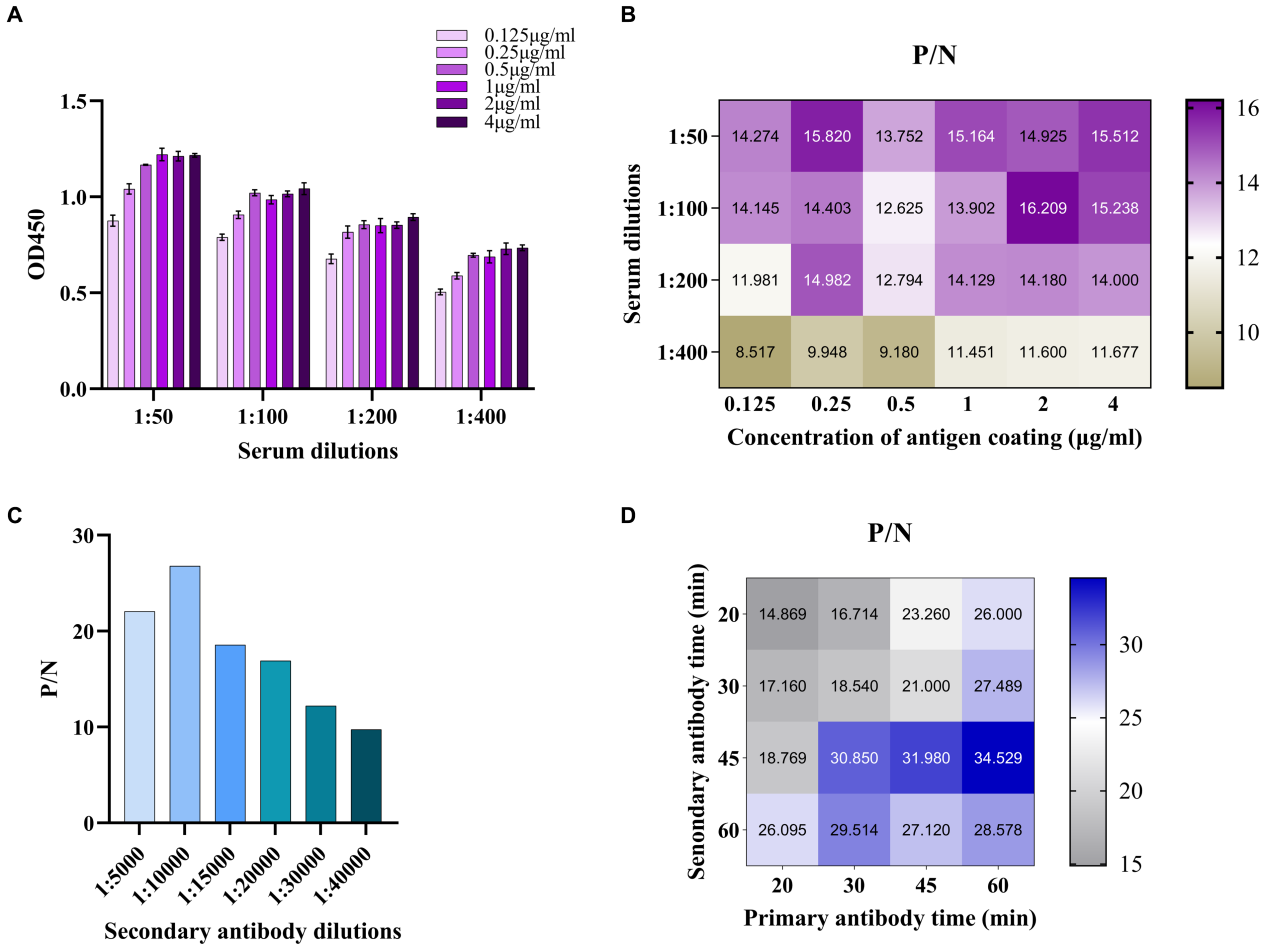
Establishment and optimization of ELISA method for antibody detection of ASFV based on p34. **(A)** The checkerboard titration was used to optimize the parameters of antigen coating concentration and antibody dilution. **(B)** The P/N value was used to determine the optimized parameters. **(C)** The optimal concentration of secondary antibody was optimized. **(D)** The P/N value was used to determine the optimal primary and secondary antibody time.

The checkerboard titration was used to optimize the parameters of antigen coating concentration and antibody dilution. The results showed that the optimal conditions for coating antigen were 2 μg/mL and 1:100 for primary antibody dilution with a maximum value of P/N ratio (Table 2; Figures 5A,B). Based on these conditions, the optimal dilution for the secondary antibody was at 1:10000 (Figure 5C) and the incubation time of the primary antibody and secondary antibody were at 37°C for 60 min and 45 min (Figure 5D). This setup was used to carry out the follow-up study. The cutoff value was determined by the sum of the mean OD450 nm value (^−^X) and three standard deviations (SDs) of negative sera. A total of 22 negative sera were used to measure and the ^−^X was 0.101 with SD 0.089. The ELISA threshold established in this study was (3SD + ^−^X) 0.368.

### Application of the ELISA for the detection of ASFV p34

**Figure 6:**
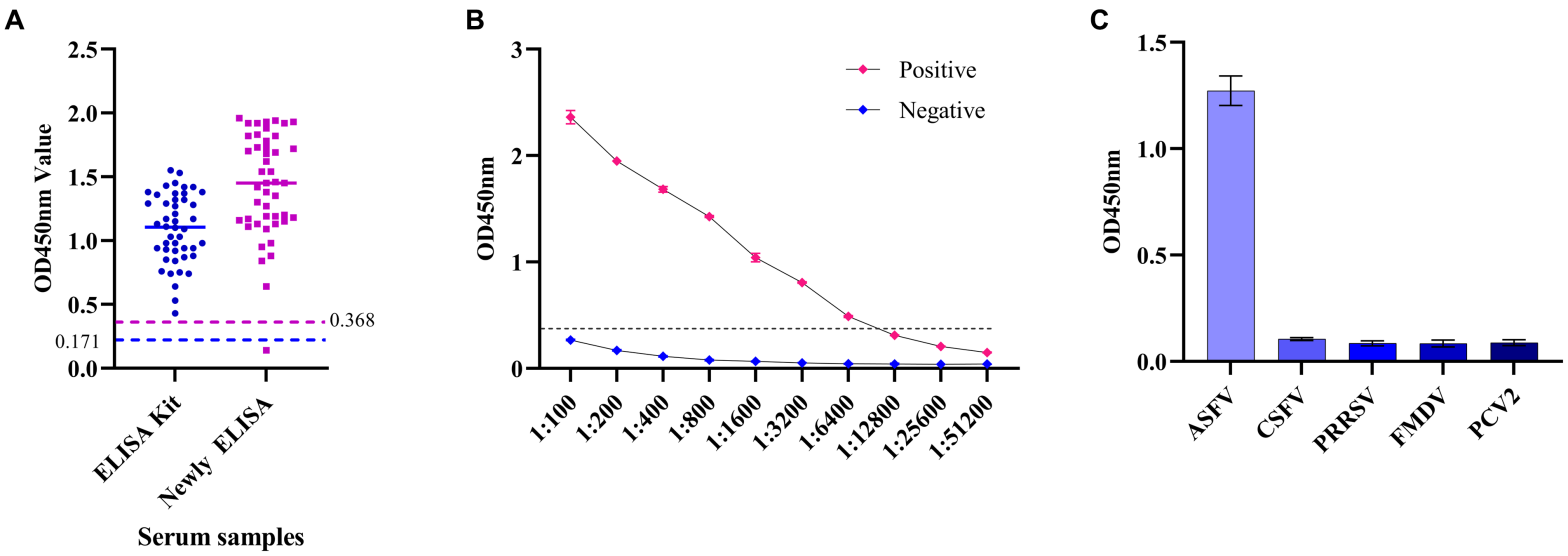
The specificity and sensitivity analysis of the established ELISA method. **(A)** The ELISA based on the p34 protein in this study was compared with commercial kits by detecting the positive serums of ASFV. **(B)** Sensitivity analysis of ELISA based on p34 protein. **(C)** Specificity analysis of ELISA based on p34 protein.

Clinical swine samples were used to verify the following analysis. Among them, the positive serum of ASFV includes genotype I and genotype II. It is proved that the established ELISA based on the p34 protein can detect two genotypes. The coincidence rate was 97.83% (45/46) with the commercial kit (Figure 6A). The ELISA can detect a minimum serum dilution of 1:6400 (Figure 6B), which could be consistent with commercially available kits (1:6,400). The results showed that the ELISA established in this study had negative reactivity with the CSFV, FMDV, PRRSV, and PCV2 (Figure 6C). In other words, there is no cross-reactivity with the above virus antibodies (Table 3).

**Table 3:** Strain sequence information used in this study.

No.	Name	NCBI accession no.
1	ASFV/pig/China/CAS19-01/2019	MN172368.1
2	ASFV/GZ201801	MT496893.1
3	ASFV/wbBS01	MK645909.1
4	ASFV/CN/2019/InnerMongolia-AES01	MK940252.1
5	ASFV/HuB20	MW521382.1
6	ASFV/Pig/Heilongjiang/HRB1/2020	MW656282.1
7	ASFV/ DB/LN/2018	MK333181.1
8	ASFV/Wuhan 2019–2	MN393477.1
9	ASFV/Wuhan 2019–1	MN393476.1
10	ASFV/China/2018/AnhuiXCGQ	MK128995.1
11	ASFV/Pig/HLJ/2018	MK333180.1

## Discussion

Up to now, African swine fever infections have been reported in more than 60 countries and regions worldwide (Clemmons et al., 2021). In 2018, the first case of ASFV was also detected in China, and the virus is lethal to domestic pigs at a rate of up to 100% (Clemmons et al., 2021), it has caused significant financial losses for the domestic pig field and poses a risk of long-term transmission (Wormington et al., 2019). In the absence of available commercial vaccines and effective therapeutic drugs, timely detection is of great significance for the prevention and control of ASF infection. Therefore, developing an economical and practical detection method has become a key goal.

Currently, ELISA, PCR, Immunofluorescence assay (IFA) colloidal gold test strips, and other techniques are frequently used for the detection of ASFV. World Organisation for Animal Health (OIE) recommends PCR and ELISA among them for the diagnosis of ASFV. There are established methods based on monoclonal antibodies to detect ASFV antigenic proteins, but because the natural strains of ASFV are continuously evolving and losing virulence, it is challenging to detect ASFV antigens through detection, which increases the risk of ASFV spreading further. It would be helpful to develop a method of diagnosing ASFV antibodies in order to resolve this conundrum.

The currently reported target proteins suitable for ASFV diagnosis mainly include structural proteins p72, p30, p54, pp62, and pp220. Among them, p72, p30, and p54 have been described for antibody detection. For example, Geng et al. developed a colloidal gold test strip based on p72 trimer labeling for the detection of ASFV antibodies. A blocking ELISA based on the p30 antibody was developed by Yu et al. to diagnose antibodies in ASFV serum (Zhao et al., 2022). Zhao et al. established a competition ELISA using p54 nanobody for the detection of antigens in ASFV. Zhao et al. (2022) established a nanoplasma biosensor with a hyperoptical transmission (EOT) effect based on the p30 protein to detect antibodies in ASFV serum. Wan et al. (2022) established antibody colloidal gold double immunochromatographic strips (ICSs) based on ASFV p30 and p72 proteins. Contrarily, this study used the cleavage product p34 of the nucleocapsid protein pp220, which makes up one-third of the ASFV virus and was chosen as a diagnostic target due to its strong immunogenicity and 100% conservation in each ASFV strain. In comparison to p72, p54, and p30, the p34 protein is a nucleocapsid protein that is easier to express and prepare in large quantities. In this research, the prokaryotic system *E. coli* was chosen to express the antigen protein. The advantage of this method is that it allows for quick and cost-effective production of gene expression products. It is also relatively simple and enables the expression of a greater number of proteins. However, a disadvantage is that the expressed proteins are not modified and may lack natural activity. Additionally, the expression system does not allow for regulation of expression time and level. Continuous expression of certain genes can be toxic to host cells and overexpression may result in non-physiological reactions. Presently, proteins are often expressed in the form of inclusion bodies, which can make product purification challenging. However, in this study, the p34 protein, which is a nucleocapsid protein, no post-translational modification required and then expressed in prokaryotic systems up to 30 mg/L medium, making it a less expensive and easier to prepare antigenic protein with advantages for the diagnosis of ASFV.

In this study, the monoclonal antibody prepared based on the p34 protein had high affinity and antibody titer and the established antibody detection ELISA method can detect 1:6400. At the same time, the product is specific and had no cross-reaction with PCV2, FMDV, CSFV, PRRSV, and other common swine infection virus antibodies.

In conclusion, ASFV p34 protein was used for the first time as a target for antibody detection. Highly active and immunogenic p34 was produced in large quantities in prokaryotic *E. coli* cells, and based on this, high-affinity and antibody titers mAb 1E6 were created and identified. MAb 1E6 recognized the 100% conserved B-cell epitope region aa 202–210. Based on this research, the established ELISA method for antibody detection of ASFV p34 has high sensitivity and specificity, which revealed a potential antigen target for diagnosis of ASFV. In short, the monoclonal antibody 1E6 prepared in this study provides useful biological materials for the study of ASFV, and the established ELISA method provides potential application value for the monitoring and control of ASFV disease.

## Data availability statement

The original contributions presented in the study are included in the article/supplementary material, further inquiries can be directed to the corresponding author.

## Ethics statement

The Henan Academy of Agricultural Sciences’ Ethics and Animal Welfare Committee granted approval for the study’s animal trials (approval code: LLSC410076). In the sterile setting, all of the little creatures were fed and given compassionate care.

## Author contributions

YT: Data curation, Formal analysis, Methodology, Resources, Software, Visualization, Writing – original draft. CL: Methodology, Writing – review & editing. JZ: Methodology, Writing – review & editing. FS: Data curation, Formal analysis, Writing – review & editing. YL: Software, Writing – review & editing. YC: Formal analysis, Writing – review & editing. XZ: Software, Writing – review & editing. HL: Methodology, Writing – review & editing. PD: Formal analysis, Writing – review & editing. EL: Data curation, Writing – review & editing. YZ: Data curation, Formal analysis, Writing – review & editing. SW: Data curation, Writing – review & editing. AW: Conceptualization, Funding acquisition, Writing – review & editing.
